# Systemic Biomarkers Do Not Discriminate Psoriasis, Atopic Dermatitis and Their Coexistence: An Equivalence-Based Cross-Sectional Analysis of 160,782 UK Biobank Participants

**DOI:** 10.3390/metabo16090632

**Published:** 2026-08-30

**Authors:** Fabiola Welter Ribeiro, Emilton Lima Junior

**Affiliations:** Postgraduate Program in Internal Medicine and Health Sciences, Department of Clinical Medicine, Federal University of Paraná, Curitiba 80060-900, Brazil; emiltonjr@ufpr.br

**Keywords:** psoriasis, atopic dermatitis, multimorbidity, immunometabolism, systemic inflammation, glycoprotein acetyls, equivalence testing, UK Biobank

## Abstract

**Highlights:**

**What are the main findings?**

- Circulating inflammatory and metabolic biomarkers did not discriminate psoriasis, atopic dermatitis or their coexistence, with 38 of 48 contrasts meeting a formal criterion for equivalence.
- Coexistence showed no biomarker synergy, and an apparent severity gradient did not persist once cardiometabolic exclusions were applied symmetrically to all groups.

**What are the implications of the main findings?**

- Systemic biomarker panels of this type cannot distinguish these conditions from one another or stratify patients carrying both diagnoses.
- Apparent gradients across dermatological groups require a symmetric comparator before they can be attributed to disease rather than to comorbidity structure.

**Abstract:**

Background/Objectives: Psoriasis and atopic dermatitis have been conceived as opposite immunological poles, yet molecular overlap and clinically recognised coexistence challenge this dichotomy. We sought to adjudicate among three architectures: overlap with no discriminating systemic signature, a shared inflammatory and metabolic continuum with additive coexistence, and distinct phenotypes with synergy. Methods: Cross-sectional analysis of a selected UK Biobank subsample of 160,782 participants, classified by International Classification of Diseases, 10th revision codes L40 and L20 as control, isolated atopic dermatitis (*n* = 12,859), isolated psoriasis (*n* = 15,146) and coexistence (*n* = 1068). Haematological indices, a biochemical and metabolomic panel including glycoprotein acetyls, and circulating inflammatory proteins were evaluated through converging evidence from ordinal effect sizes, equivalence testing at a margin equivalent to an area under the receiver operating characteristic curve of 0.574, quantile regression with interaction terms, permutational multivariate analysis of variance, supervised classification and mixture modelling. Results: Effect sizes were negligible for 46 of 48 contrasts against a comorbidity-free comparator, and 38 of 48 met formal equivalence. None of the 16 interaction terms survived false discovery rate control. Multivariate separation was detectable but explained 0.6% of dispersion, classification reached an area under the curve of 0.57, and latent classes bore no correspondence to clinical diagnosis. When cardiometabolic exclusions were applied symmetrically to all four groups, every contrast met equivalence, and the ordering of the dominant factor did not persist. Polygenic risk scores confirmed that the coded groups carry the expected genetic architecture. Conclusions: Within the systemic biomarkers evaluated these conditions are not discriminable, and coexistence is not an emergent phenotype.

## 1. Introduction

Chronic inflammatory skin diseases are no longer understood as processes confined to the integument, but as systemic conditions in which cutaneous inflammation is accompanied by metabolic, haematological and cardiovascular repercussions measurable in the circulation [1,2]. Psoriasis is the most established paradigm of this shift: the IL-23/IL-17 axis that sustains the psoriatic plaque also participates in endothelial dysfunction, insulin resistance and adipose-tissue inflammation, articulating the concept of the psoriatic march, which links the skin to accelerated atherosclerosis [1,2]. Consistently, psoriasis is associated with higher odds of metabolic syndrome in syntheses of observational studies [3]. Atopic dermatitis, classically anchored in type 2 immunity, exhibits its own systemic inflammatory signatures, with type 2 and type 17 co-activation detectable in peripheral blood [4,5].

Historically, psoriasis and atopic dermatitis were regarded as opposite immunological poles. This dichotomy has been progressively qualified as molecular studies reveal overlapping mediators and a shared inflammatory core, and the question of whether they represent two diseases or one spectrum has been posed explicitly [6,7]. The parallel with asthma is instructive: what was assumed to be a single entity proved to be a set of phenotypes and endotypes, now discriminated through supervised classification and unsupervised clustering of biomarkers [8]. The same question of nosological architecture remains open for the psoriasis and atopic dermatitis pair, particularly when both coexist in the same individual [9].

Coexistence was long considered uncommon, and the mechanistic rationale for that expectation is the reciprocal antagonism between the type 17 and type 2 axes described in patients carrying both diagnoses [10]. Where it occurs, it constitutes a natural experiment of singular value [9]. If the two diseases represent genuinely distinct phenotypes, an individual harbouring both should display a signature that additively combines the deviations of each or exceeds it. If they represent positions along a single spectrum, coexistence should behave as a graded intensification of a common axis. If neither, the systemic compartment may simply be uninformative about which of the two diseases a patient has. Distinguishing these architectures bears on cardiometabolic risk stratification, elevated both in severe atopic dermatitis [11] and across the psoriatic spectrum [12].

Population cohorts with deep molecular phenotyping, such as UK Biobank [13], allow this question to be addressed by integrating lipid and hepatic profiles, circulating inflammatory proteins quantified by proximity extension assays [14], metabolomic markers of systemic inflammation obtained by nuclear magnetic resonance such as glycoprotein acetyls [15], and inflammatory indices derived from the complete blood count. Glycoprotein acetyls, in particular, is a stable marker of low-grade inflammation and a predictor of cardiometabolic risk from early ages [16]. The aim of this exploratory analysis is to adjudicate among the three architectures set out above.

## 2. Materials and Methods

### 2.1. Design, Nature of the Analysis and Reporting

Unlike the pre-specified statistical plan underpinning the project, the present analysis was conducted with direct access to the outcomes and should be interpreted as exploratory and hypothesis-generating, not confirmatory. The results reported here do not replace the formal, pre-registered execution within the governed UK Biobank environment, which they are intended to inform. Reporting follows, where applicable, the STROBE recommendations for cross-sectional observational studies [17]; the completed checklist is provided as Supplementary Materials.

UK Biobank holds ethical approval from the North West Multi-centre Research Ethics Committee (REC reference 11/NW/0382), and all participants provided informed electronic consent. This research was conducted under UK Biobank application number 358626.

### 2.2. Case Ascertainment and Group Definition

Health outcomes in UK Biobank are not collected as a purpose-built clinical registry. They are assembled by linkage of the baseline assessment to national health datasets, and the resulting phenotypes therefore carry the properties of the administrative systems that generated them [13,18]. Four sources contribute. Hospital inpatient activity is drawn from Hospital Episode Statistics for England, the Scottish Morbidity Records and the Patient Episode Database for Wales, coded in ICD-10 across primary and secondary diagnostic positions. Mortality data come from the national death registries. Primary care records, available for approximately half of the cohort, are coded in Read version 2 and Clinical Terms version 3. Self-reported conditions were captured at the assessment centre through a nurse-led verbal interview.

For a defined set of conditions, UK Biobank harmonises these sources into first-occurrence fields, in which each three-character ICD-10 code is accompanied by a date of first report and by a field identifying which source contributed that first record [18]. Codes originating in primary care and in self-report are mapped to their ICD-10 three-character equivalents before harmonisation. Three consequences follow, and each constrains interpretation. The resulting phenotype is composite, in that a participant is classified as affected if any source contributed a record. Aggregation occurs at the three-character level so that L40 subsumes L40.0 through L40.9, including arthropathic psoriasis, and L20 subsumes L20.0, L20.8 and L20.9. Finally, the fields are constructed independently for each code and impose no mutual exclusivity, so a participant may legitimately carry records for both L40 and L20.

Group definition followed directly from this structure. Psoriasis was defined by a first-occurrence record for L40 and atopic dermatitis by a first-occurrence record for L20. Four mutually exclusive groups were constituted from 160,782 participants drawn from 501,979 by the presence of GLP-1 receptor assay data or a record of any of six index conditions (L40, L20, E66, I21, I48, I64): control, defined by the absence of both codes; isolated atopic dermatitis; isolated psoriasis; and coexistence, defined by the presence of both codes in the same individual. Throughout this article, coexistence denotes the presence of concurrent coded records rather than clinically adjudicated concurrent disease; where the clinical entity is intended, we write concurrent psoriasis and atopic dermatitis. The derivation is summarised in Figure S1.

Because the subsample is enriched for cardiometabolic disease, a control defined solely by the absence of L40 and L20 remains contaminated by obesity, myocardial infarction, atrial fibrillation and stroke. A restricted control, additionally free of E66, I21, I48 and I64 (*n* = 31,257), was therefore adopted as the primary comparator, and a broad control comprising all participants without L40 or L20 (*n* = 131,709) was retained as a sensitivity analysis. Because these comorbidities are zero by definition in the restricted control, covariate adjustment for them is not estimable within that comparison; a sensitivity analysis applying the same exclusions symmetrically to all four groups is reported in Section 3.11.

### 2.3. Variables and Biomarker Panels

Three layers with distinct coverage were analysed. The haemato-inflammatory panel, comprising the neutrophil-to-lymphocyte ratio, platelet-to-lymphocyte ratio, monocyte-to-lymphocyte ratio, leucocyte-to-lymphocyte ratio, monocyte-to-HDL ratio, systemic immune–inflammation index and systemic inflammation response index, had coverage close to 95%; such ratios have been studied as correlates of systemic inflammation and subclinical atherosclerotic cardiovascular disease in psoriasis [19]. The biochemical and metabolomic panel comprised total cholesterol, LDL, VLDL, HDL, triglycerides, alanine aminotransferase, aspartate aminotransferase, glycated haemoglobin and glycoprotein acetyls; coverage ranged from 55.6% for the nuclear magnetic resonance metabolomics subset, which includes glycoprotein acetyls, to 94% for the routine biochemistry.

Olink inflammatory proteins (IL-6, IL-17A, IL-17C, IL-17F, IL-18, IL-12, IL-4, IL-13, IL-31, FCER1A and FCER2), expressed as relative normalised protein expression values on a log scale [14], were available for approximately 29% of the analytic sample. Coverage was not uniform across groups: it was 32.3% in the control group, 13.9% in atopic dermatitis, 10.8% in psoriasis and 15.3% in coexistence, corresponding to 42,558, 1786, 1631 and 163 participants respectively. The proteomic subset is therefore differentially ascertained as well as small in the coexistence group, and this arm is treated as exploratory throughout and excluded from the decision synthesis.

### 2.4. Covariates

The adjustment set comprised age, sex, body mass index, ethnicity (white versus other), statin use identified through text analysis of medication codes, and smoking. The smoking covariate is a derived binary indicator marking 11.7% of the analytic sample, a prevalence consistent with current rather than ever-smoking in this cohort; former tobacco exposure is therefore not captured, and residual confounding by past smoking is not addressed.

Household income was available for 86.5% of the analytic sample, physical activity for 95.0% and television viewing time for 96.3%. These were not used in the primary models but were added to the adjustment set in the extended sensitivity analysis reported in Section 3.11. Dietary pattern, alcohol intake and the Townsend deprivation index were not used. Fasting time and assessment centre were not present in the extraction, and the age field corresponded to attained age rather than age at recruitment.

Statin use was more frequent in the broad control (26.7%) than in psoriasis (18.7%) or atopic dermatitis (15.4%), reflecting the greater cardiometabolic burden and screening intensity of the group without skin disease. This pattern is capable of masking or reversing lipid deviations and was explicitly considered in interpretation.

### 2.5. Pre-Processing

Each biomarker was standardised to a z-score using the median and interquartile range of the restricted control as reference so that deviations were expressed in comparable, summable units, a condition necessary for the additivity test. Scores were winsorised at plus or minus five standard deviations. Multivariate analyses were conducted on complete cases with the effective number reported, and multiple imputation by chained equations was performed as a confirmatory sensitivity analysis (Section 3.11).

### 2.6. Software and Code Availability

The analyses reported in the present version were carried out, or independently re-executed and verified, in Python 3.12.3 using NumPy 2.4.4, SciPy 1.17.1, pandas 3.0.2, statsmodels 0.14.6, scikit-learn 1.8.0, Matplotlib 3.10.8 and openpyxl 3.1.5. During revision, the descriptive characterisation, the ordinal effect sizes and equivalence tests, the principal component analysis and ordination, the permutational analyses of variance and dispersion, the mixture modelling and the supervised classification were re-executed from the source dataset in this environment. Agreement with the originally submitted values was obtained for the proportion of multivariate variance explained, the dispersion statistic, the range of effect sizes and the descriptive characterisation; the quantities that did not reproduce are identified and resolved where they are reported, in Section 3.5, Section 3.6 and Section 3.7.

The three post hoc analyses reported in Section 3.9 and Section 3.11, namely the comparison of observed against expected frequency of coexistence, the polygenic validation of the group definitions and the symmetric cardiometabolic exclusion, together with the extended covariate adjustment and the generation of all figures, were performed in the same environment.

No novel software was developed. The analysis scripts supporting the results reported here are available from the corresponding author upon reasonable request. Generative artificial intelligence was used to assist in the execution and tabulation of these analyses, as disclosed in Section 2.10.

### 2.7. Analytical Architecture and Decision Framework

The analyses reported below draw on methods of different families: ordinal effect sizes, quantile regression, permutational multivariate analysis of variance, supervised classification and mixture modelling. This heterogeneity is deliberate and follows from the structure of the question rather than from an accumulation of techniques.

The three competing accounts are not distinguished by the size of any single quantity. They are distinguished by a pattern across several quantities, and each quantity requires a different instrument. Effect size can establish that differences are small but cannot establish whether small differences are ordered. Ordination testing can establish ordering but cannot establish whether the underlying structure is continuous or discrete. Mixture modelling can establish discreteness but cannot quantify magnitude. Classification can establish whether group differences suffice to identify individuals, which is the question of clinical interest, and no measure of aggregate separation answers it. A study using only one of these methods could not reach a defensible conclusion regardless of its sample size. The framework is therefore best described as converging evidence rather than as a multivariate analysis. Each method was assigned in advance to a target, an estimand and a decision criterion (Table 1).

Two features of this framework govern how the results should be read. First, the criteria are not symmetric in what they can establish. Failure to detect separation is compatible with H0 and H1 alike; only equivalence testing and ordination testing distinguish them. Conversely, separation of negligible magnitude is evidence against H2 whatever its statistical significance, so magnitude is reported alongside significance throughout, and significance alone is treated as uninformative at this sample size.

Second, the criteria differ in their vulnerability to bias. Unmeasured confounding, comparator construction and outcome misclassification tend to generate apparent differences between groups and to attenuate nothing. Criteria whose satisfaction depends on observing a difference, namely separation, discrimination, synergy and ordination, are therefore vulnerable to these biases, whereas criteria whose satisfaction depends on observing equivalence are conservative with respect to them. This asymmetry is examined empirically in Section 3.11.

The architecture above was specified before analysis. Three analyses reported here were not and are identified as post hoc: the comparison of observed against expected frequency of coexistence under independence of the two code families, the genetic validation of the group definitions using polygenic risk scores, and the sensitivity analysis applying cardiometabolic exclusions symmetrically. The first two were added in response to peer review, and the third was performed at the explicit suggestion of a reviewer.

### 2.8. Executed Analysis Plan

Magnitude and uncertainty, rather than significance in isolation, guided interpretation. Effect sizes were estimated by Cliff’s delta [20], with bootstrap confidence intervals and the established negligibility threshold of |δ| < 0.147 [21]. Cliff’s delta stands in exact algebraic relation to the area under the receiver operating characteristic curve, since the area equals one half of the quantity delta plus one; the margin of 0.147 therefore corresponds to an area of 0.574, meaning that a randomly chosen patient exceeds a randomly chosen control in 57.4% of comparisons against 50% by chance. A biomarker performing at that level cannot stratify, triage or classify individual patients, and this is the operational sense in which such differences are described as negligible.

Additivity was tested by median quantile regression with psoriasis and atopic dermatitis indicators and their interaction term, adjusted for the available covariates, and by MANOVA of the interaction. Separation was assessed by PERMANOVA with Euclidean distance [22] and by classifiers with cross-validation and null validation by permutation. Latent structure was investigated by principal component analysis and Gaussian mixture modelling, with agreement measured by the adjusted Rand index and normalised mutual information. Ordination was tested by extracting the dominant factor and applying the Jonckheere–Terpstra trend test. Overlap was tested affirmatively by equivalence testing against the margin |δ| = 0.147 [23]. False discovery rate control followed the Benjamini–Hochberg procedure by family [24].

### 2.9. Additional Sensitivity Analyses

The equivalence margin was re-evaluated at three values (|δ| = 0.11, 0.147 and 0.20, corresponding to areas under the curve of 0.555, 0.574 and 0.600), and the empirical distribution of |δ| was examined. Homogeneity of multivariate dispersions was tested by PERMDISP. Additivity was re-examined at the 0.75 and 0.90 quantiles. Redundancy among the leucocyte ratios was quantified by the Spearman correlation and by the variance explained by their first principal component. The role of immunomodulatory and biologic therapies was assessed by excluding participants using these agents. Cardiometabolic exclusions were applied symmetrically to all four groups. The adjustment set was extended to include household income, physical activity and television viewing time. Multiple imputation by chained equations was performed as a confirmatory analysis. Finally, the smallest detectable interaction effect was estimated given the size of the coexistence group.

### 2.10. Use of Generative Artificial Intelligence

Generative artificial intelligence tools were used to improve the language, readability and formatting of author-written text and, during revision, to assist with the execution and tabulation of the additional analyses requested by peer review, all of which were specified, reviewed and verified by the authors. Grammarly Pro was used for additional grammar checking. Generative artificial intelligence was not used for study design, data collection or interpretation of the data. All outputs were reviewed and edited by the authors, who take full responsibility for the final content.

## 3. Results

### 3.1. Baseline Characterisation

From the restricted control to coexistence, a progressive increase was observed in body mass index (26.2 to 27.9 kg/m^2^), statin use (11.6% to 20.4%), the systemic inflammation response index (0.92 to 1.06) and the neutrophil-to-lymphocyte ratio (2.12 to 2.29), with a concomitant reduction in HDL (1.43 to 1.34 mmol/L). The prevalence of obesity, myocardial infarction and atrial fibrillation rose from zero, by definition, in the restricted control to 19.2%, 6.7% and 9.7% in coexistence (Table 2).

### 3.2. Univariate Effect Sizes

**Figure 1:**
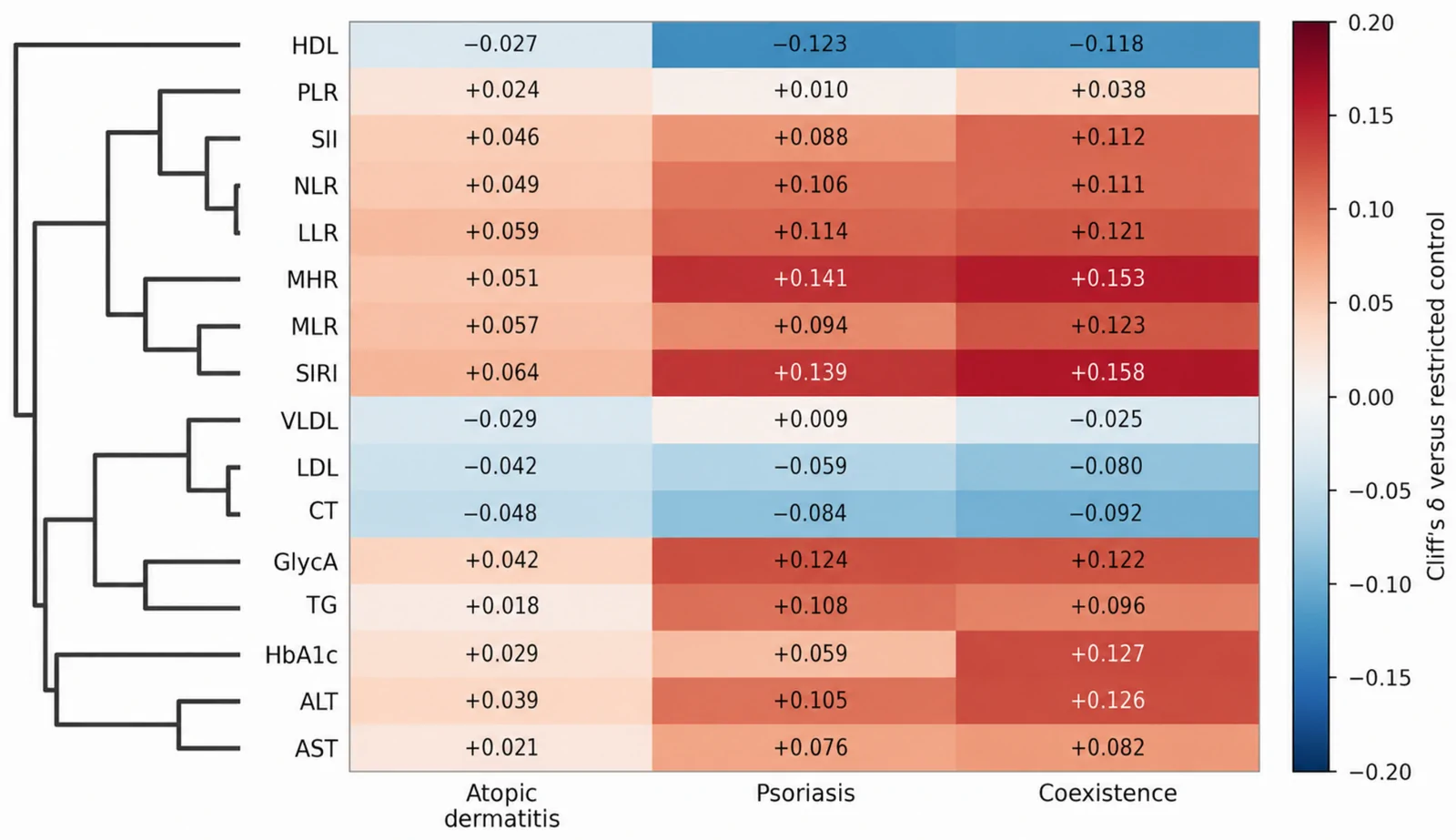
Effect sizes and hierarchical clustering of biomarkers. The cells show Cliff’s delta for each disease group against the restricted control, with positive values and warm tones indicating elevation relative to control. The dendrogram was computed from the Spearman correlation structure of the biomarker panel in the analytic sample. ALT, alanine aminotransferase; AST, aspartate aminotransferase; CT, total cholesterol; GlycA, glycoprotein acetyls; HbA1c, glycated haemoglobin; HDL, high-density lipoprotein; LDL, low-density lipoprotein; LLR, leucocyte-to-lymphocyte ratio; MHR, monocyte-to-HDL ratio; MLR, monocyte-to-lymphocyte ratio; NLR, neutrophil-to-lymphocyte ratio; PLR, platelet-to-lymphocyte ratio; SII, systemic immune–inflammation index; SIRI, systemic inflammation response index; TG, triglycerides; VLDL, very-low-density lipoprotein.

Against the restricted control, Cliff’s deltas of the robust panel ranged from −0.12 to 0.16, with 46 of 48 contrasts classified as negligible. Hierarchical clustering (Figure 1) separated an inflammatory and metabolic block, encompassing the systemic inflammation response index, monocyte-to-HDL ratio, glycoprotein acetyls, alanine aminotransferase, glycated haemoglobin and leucocyte ratios, from a lipid block with reductions in HDL, LDL and total cholesterol. Isolated atopic dermatitis showed systematically smaller deviations. The largest absolute values occurred for the systemic inflammation response index and the monocyte-to-HDL ratio in coexistence (δ = 0.158 and 0.153) and in psoriasis (δ = 0.139 and 0.141); only the two coexistence values exceeded the negligibility threshold (Table 3).

### 3.3. Circulating Inflammatory Proteins

In the proteomic subset, isolated psoriasis showed the largest deviations in IL-6 (δ = 0.20; 95% CI 0.17 to 0.23) and IL-17A (δ = 0.16; 95% CI 0.13 to 0.19), with IL-17C, IL-17F, IL-18 and IL-12 elevated to a lesser degree. Type 2 pathway markers (IL-4, IL-13 and IL-31) remained close to zero. These estimates are reported as hypothesis-generating. IL-4, IL-13 and IL-31 circulate at or near the lower limit of quantification of the assay in unselected populations, and lesional skin is the compartment in which type 2 immunopathology in atopic dermatitis is expressed; the absence of a peripheral signal is therefore uninformative about tissue-level biology rather than evidence against it. Given the differential coverage documented in Section 2.3, no hypothesis is adjudicated on proteomic evidence.

### 3.4. Additivity Versus Synergy

After adjustment, none of the sixteen psoriasis by atopic dermatitis interaction terms reached significance under false discovery rate control (none of 16; smallest q = 0.51 for glycated haemoglobin). The global multivariate interaction test was statistically significant but of trivial magnitude (Pillai trace 0.0005; *p* = 0.003), corresponding to less than 0.1% of the multivariate variance. The coexistence profile is therefore approximately the sum of the deviations of the isolated diseases, with no evidence of synergy.

### 3.5. Multivariate Separation and Discrimination

Permutational multivariate analysis of variance detected global separation among the four groups, but the clinical label explained only 0.6% of the multivariate dispersion (pseudo-F = 52.0 on 26,977 complete cases; *p* = 0.005). The value of the pseudo-F statistic reported in the previously submitted version, 3.38, was obtained on a random subsample rather than on the full analytic sample and is superseded here; the proportion of variance explained is identical under both, and it is that proportion, rather than the test statistic, that carries the interpretation. Classifiers achieved a multiclass area under the receiver operating characteristic curve of 0.57 for penalised logistic regression and 0.56 for random forest, against 0.49 under a permutation null, indicating discriminative ability marginally above chance but without practical value. These values supersede the areas of 0.54 and 0.53 reported in the previously submitted version, which were obtained on the same random subsample as the superseded pseudo-F statistic; the revised estimates are marginally less favourable to the conclusion drawn here and are retained for that reason. This value lies at or below the equivalence margin expressed on the same scale (0.574), so the classification and equivalence analyses are measuring the same quantity and agreeing.

**Figure 2:**
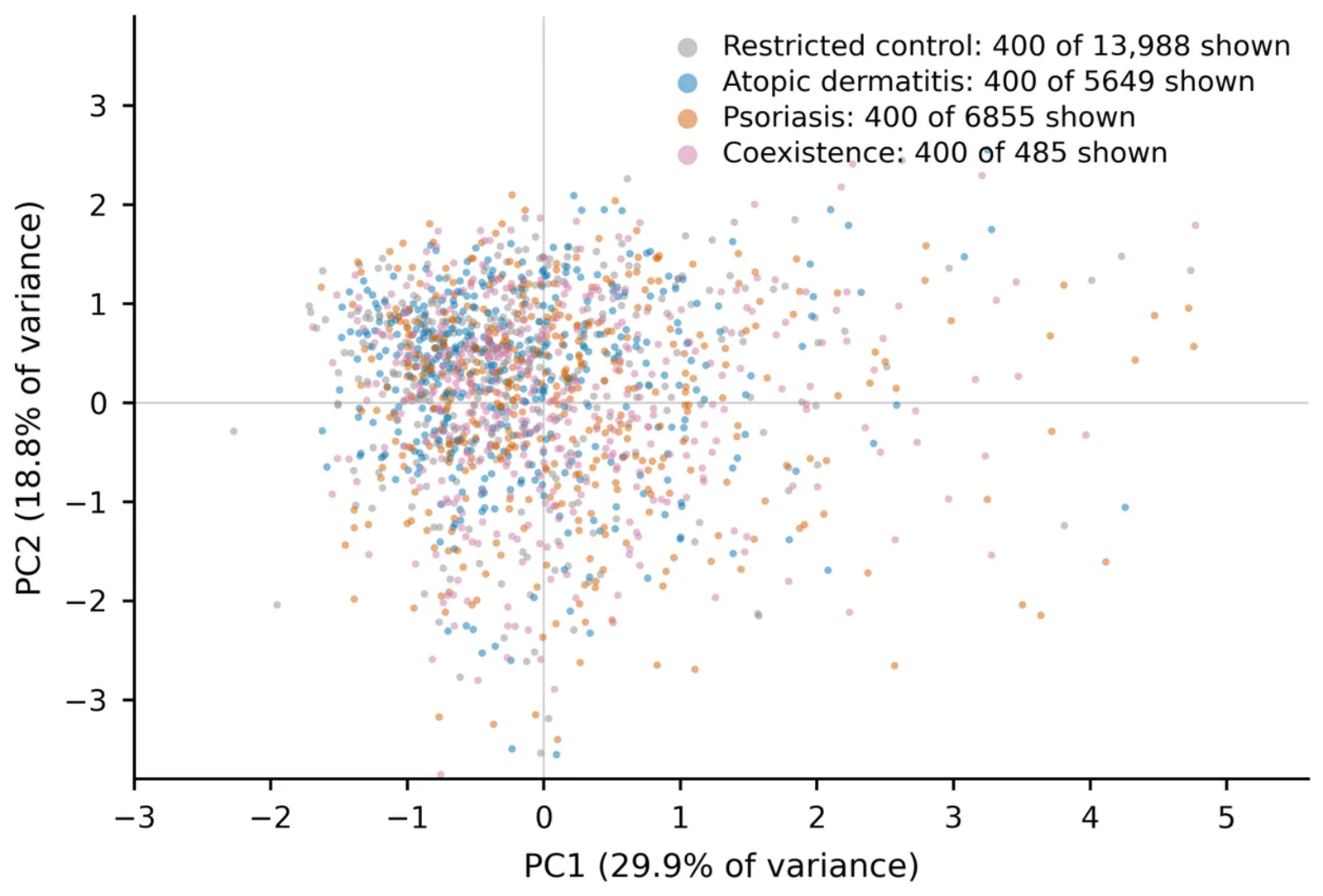
Projection of standardised biomarkers onto the first two principal components. The points are a random sample of each group, drawn to prevent the control group from obscuring the smaller groups; the number displayed and the total available are given for each group. PC1 and PC2 explain 29.9% and 18.8% of the variance of the 16-marker panel.

The principal component projection (Figure 2) showed extensive overlap. Multivariate dispersion was not homogeneous across groups (F = 98.1; *p* < 0.001), with the median distance to the centroid increasing from the restricted control (2.90) to atopic dermatitis (3.01), psoriasis (3.19) and coexistence (3.20). The dispersion statistic is unchanged from the previously submitted version, whereas the distances themselves are those of the re-executed ordination and are numerically smaller, with their ordering across groups being identical. Part of the separation detected is therefore attributable to heterogeneity of dispersion rather than to displacement of group centroids, which further weakens any reading of discrete phenotypes.

### 3.6. Unsupervised Structure

Gaussian mixture modelling selected four latent classes on the Bayesian information criterion. Agreement between these classes and clinical diagnoses was essentially nil (adjusted Rand index of 0.011; normalised mutual information of 0.005). These values were obtained in the re-execution described in Section 2.6 and supersede the adjusted Rand index of 0.004 and the normalised mutual information of 0.007 reported in the previously submitted version; on either estimate the agreement is indistinguishable from none. Although the data possess internal structure, that structure does not correspond to the separation among control, atopic dermatitis, psoriasis and coexistence.

### 3.7. Ordination

**Figure 3:**
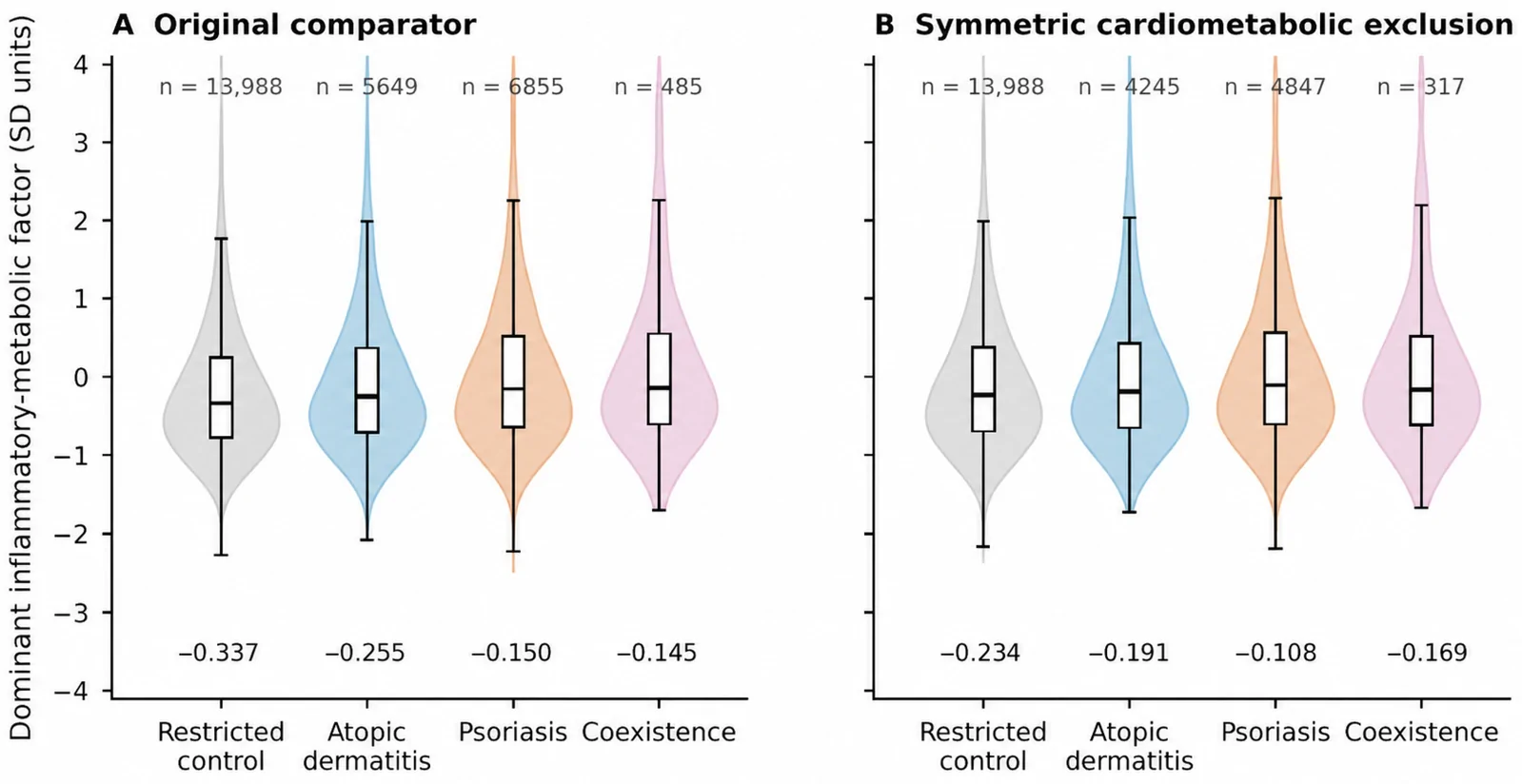
Distribution of the dominant inflammatory-metabolic factor by group. (**A**) Original comparator. (**B**) After the same cardiometabolic exclusions are applied symmetrically to all four groups. The violins show the full distribution, the boxes the interquartile range and the horizontal lines the median; the value beneath each group is its median, and the group sizes are given above each violin. Under symmetric exclusion the coexistence group falls below isolated psoriasis, and the monotonic ordering does not persist.

The dominant inflammatory and metabolic factor explained 29.9% of the variance and was anchored chiefly in leucocyte ratios, with a smaller contribution from lipid markers. These indices are recombinations of the same three or four leucocyte counts and are therefore strongly collinear, so the prominence of the haematological block within this factor is in substantial part arithmetic rather than biological; the redundancy is quantified in Section 3.11. Under the original comparator the medians of this factor were ordered from the restricted control (−0.337) to atopic dermatitis (−0.255), psoriasis (−0.150) and coexistence (−0.145), with a significant Jonckheere–Terpstra trend. The factor scores were recomputed in the re-execution described in Section 2.6 and are numerically different from the medians of −0.56, −0.39, −0.16 and −0.15 reported in the previously submitted version, while the ordering of the groups, the overlap between psoriasis and coexistence and the trend statistic are unchanged. Psoriasis and coexistence occupied the upper end of the observed axis together and were not separable from one another, with the difference in medians being smaller than the precision at which the statistic is reported. As shown in Section 3.11, this ordering does not persist once cardiometabolic exclusions are applied symmetrically, and the trend statistic principally reflects the separation of the control group from the three disease groups rather than graded ordering among the diseases (Figure 3).

### 3.8. Confirmation of Overlap by Equivalence

**Figure 4:**
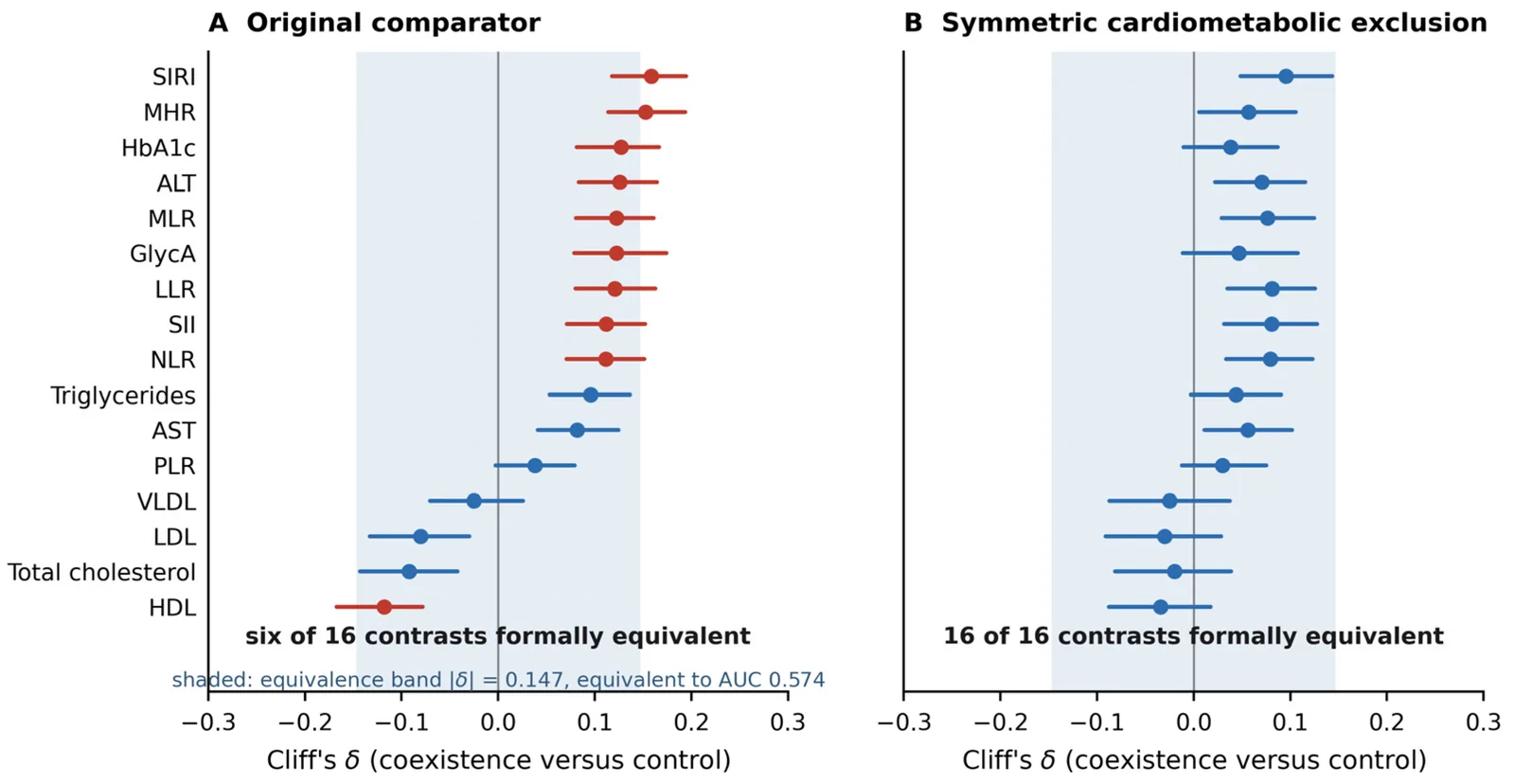
Cliff’s delta for the coexistence group against the control, with 95% bootstrap confidence intervals and the equivalence band. The shaded band marks the margin |δ| = 0.147, equivalent to an area under the curve of 0.574, and the vertical line marks δ = 0. Colour encodes the equivalence decision and not any property of the biomarker itself: blue denotes contrasts whose entire confidence interval lies within the band and which therefore meet the criterion for formal equivalence, and red denotes contrasts whose interval extends beyond the margin. Because the criterion is applied to the interval and not to the point estimate, a contrast may have a point estimate inside the band and still be shown in red when its interval crosses the margin. (**A**) Original comparator, in which the coexistence group is compared with the restricted control and 6 of 16 contrasts meet the criterion. (**B**) After symmetric cardiometabolic exclusion, in which every contrast examined meets the criterion. Biomarkers are ordered by effect size under the original comparator, and the order is preserved in both panels.

Under the margin |δ| = 0.147, 38 of 48 contrasts were statistically equivalent to the restricted control, rising to 47 of 48 under the broad control. Forty-six of 48 contrasts had point estimates below the margin, whereas 38 met formal equivalence; these figures are not contradictory, since equivalence testing requires the entire confidence interval to lie within the margin, and the contrasts that failed did so through imprecision rather than magnitude. The proportion meeting equivalence was margin-dependent: 26 of 48 at |δ| = 0.11 (area under the curve 0.555), 38 of 48 at 0.147 (0.574) and 48 of 48 at 0.20 (0.600). The empirical distribution of |δ| (median, 0.081; 90th percentile, 0.126; maximum, 0.158) places the adopted margin just above the 90th percentile of observed effects. The qualitative conclusion of negligible discrimination holds across the range because the point estimates themselves are small (Figure 4).

### 3.9. Validity of the Group Definitions

Two post hoc analyses examined whether the coded groups behave as the diseases they name.

First, the observed frequency of coexistence was compared with that expected under independence of the two code families. Among 160,782 participants, 16,214 carried any L40 record, and 13,927 carried any L20 record. Under independence, 1404 would be expected to carry both; 1068 were observed. The observed-to-expected ratio was 0.76, with an odds ratio of 0.72 (95% CI 0.68 to 0.77; χ^2^ = 97.8; *p* < 0.001). Coexistence is therefore recorded less often than chance would predict, a direction incompatible with permissive coding or indiscriminate double labelling, which would inflate the count above independence.

**Figure 5:**
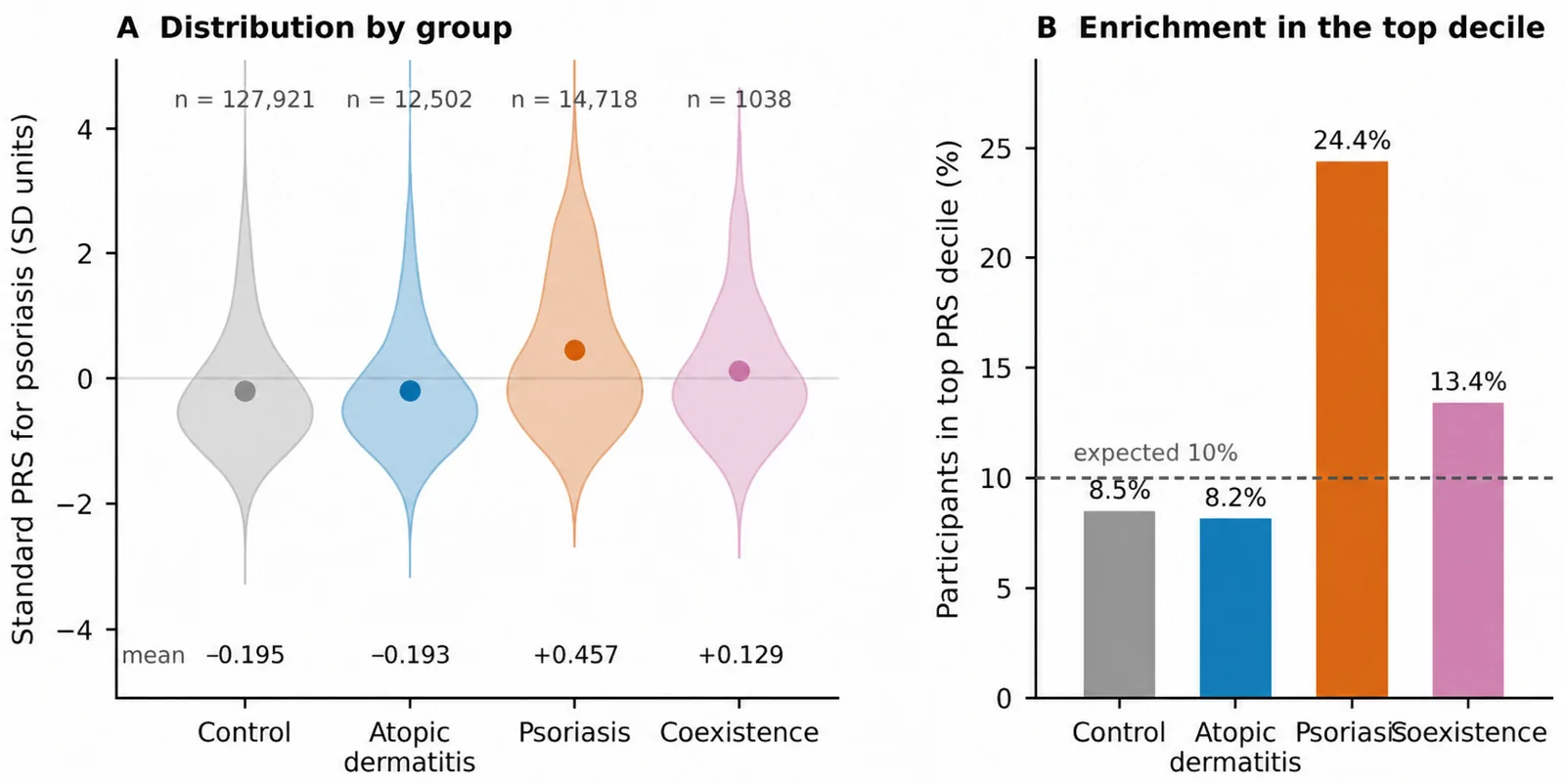
Genetic validation of the group definitions. (**A**) Distribution of the standard polygenic risk score for psoriasis by group, with the violins showing the full distribution and the markers showing the group mean with 95% confidence interval. (**B**) Proportion of each group falling in the top decile of the cohort-wide score distribution, against the 10% expected under no enrichment. PRS, polygenic risk score.

Second, standard polygenic risk scores for psoriasis, available for 97.2% of the analytic sample, were compared across groups (Figure 5). The isolated atopic dermatitis group was indistinguishable from the control group (δ = −0.005; *p* = 0.38), serving as a negative control. The coexistence group carried psoriasis polygenic loading exceeding both the control group (*p* = 6 × 10^−25^) and the isolated atopic dermatitis group (*p* = 7 × 10^−23^) but attenuated relative to isolated psoriasis (*p* = 7 × 10^−16^). The mean displacement from the control was +0.324 standard deviations for coexistence against +0.652 for isolated psoriasis, implying under a simple mixture model that approximately half the coexistence group carries loading consistent with true psoriasis. An independent clinical check converged on the same estimate: arthropathic psoriasis, captured by L40.5 and M07, occurred in 9.9% of the isolated psoriasis group and 5.6% of the coexistence group, a ratio of 0.57. By contrast, asthma and allergic rhinitis were recorded in 23.6% and 21.2% of the coexistence group against 23.9% and 21.8% of the isolated atopic dermatitis group, that is, essentially identically.

### 3.10. Decision Synthesis

No criterion supported distinct immunometabolic phenotypes with synergy. The criterion for affirmative overlap was satisfied for the large majority of contrasts under the primary comparator and for all contrasts examined under symmetric exclusion. The one criterion that would have distinguished a shared continuum from the absence of a signal, monotonic ordination, is inconclusive (Table 4).

### 3.11. Sensitivity Analyses

Symmetric cardiometabolic exclusion. Removing E66, I21, I48 and I64 from all four groups rather than from the control alone left 31,257 controls, 9693 with atopic dermatitis, 10,729 with psoriasis and 727 with coexistence. Under this comparator all 48 contrasts examined were negligible, and all 48 met formal equivalence, against 46 and 38 respectively under the original design. Metabolic contrasts attenuated by roughly half, with glycated haemoglobin in coexistence falling from +0.127 to +0.038 and glycoprotein acetyls in psoriasis from +0.124 to +0.083 on the control-referenced scale. The dominant factor medians became −0.234, −0.191, −0.108 and −0.169 so that coexistence fell below isolated psoriasis and the monotonic ordering did not persist. A material component of the gradient reported under the original comparator therefore reflects comparator construction rather than dermatological disease.

Extended adjustment. Adding household income, physical activity and television viewing time to a model already adjusted for age, sex, body mass index and ethnicity did not materially alter any estimate. The largest change was for glycoprotein acetyls in coexistence, from +0.053 to +0.017 standard deviations of the rank-normalised biomarker, and all coefficients remained at or below 0.17 standard deviations.

Treatment. Excluding participants using immunomodulatory or biologic therapies, more frequent in psoriasis (5.0%) and coexistence (3.3%) than in atopic dermatitis (0.8%) or controls (1.0%), did not materially alter effect sizes.

Missing data. Multiple imputation by chained equations reproduced the complete-case findings for the multivariate and equivalence analyses and reconciles the difference between the trend statistics obtained in the complete-case analysis and after exclusion of treated participants, which arises from differing effective sample sizes rather than from a change in the underlying pattern.

Distributional location and power. None of the sixteen interaction terms was significant at the median (smallest *p* = 0.094), and the interaction remained non-significant at the 0.75 and 0.90 quantiles, with a single nominal signal for glycated haemoglobin at the 0.90 quantile (*p* = 0.013) that did not withstand multiplicity correction. Given the size of the coexistence group, the smallest detectable interaction effect at 80% power was approximately 0.09 standard deviations, bounding the magnitude of any undetected synergy.

Collinearity. The leucocyte ratios anchoring the dominant factor were strongly correlated with one another (NLR and LLR ρ = 0.98; NLR and SII ρ = 0.85; LLR and SII ρ = 0.83), and the first principal component of the seven leucocyte-derived indices alone accounts for 65.1% of their variance, against 29.9% for the first component of the full 16-marker panel. The dominance of a single component within the haematological block is therefore in substantial part arithmetic rather than biological, since these indices are recombinations of the same three or four leucocyte counts.

## 4. Discussion

The principal finding of this analysis is negative and, we would argue, informative because of the size of the sample in which it was obtained. Across a panel of haematological, biochemical and metabolomic biomarkers measured in 160,782 participants, psoriasis, atopic dermatitis and their coexistence could not be discriminated from one another or from a comorbidity-free comparator. Separation was statistically detectable but explained 0.6% of multivariate dispersion; individual-level classification performed at an area under the curve of 0.57; latent structure in the data bore no relation to clinical diagnosis; and the large majority of contrasts met a formal criterion for equivalence.

It is important to state what this does not establish. Failure to discriminate is not demonstration of identity. These results do not show that psoriasis and atopic dermatitis constitute a single biological or nosological continuum; they show that the systemic biomarkers evaluated carry very limited information about which of these conditions a patient has. Whether this reflects genuinely shared systemic biology or the insensitivity of circulating markers to inflammation compartmentalised in the skin cannot be resolved by the present design, and the two explanations have quite different implications.

The absence of a psoriasis by atopic dermatitis interaction is the most robust positive statement available from these data. Coexistence is approximately the sum of its parts, not an emergent state, and the bound on any undetected synergy is approximately 0.09 standard deviations.

### 4.1. Validity of the Case Definitions

The coexistence group is the analytical pivot of this study and also its most exposed construct. For psoriasis, external validation is available and favourable: composite and self-reported definitions in UK Biobank have been shown to recover the expected genetic architecture rather than behaving as noise [25], and the present definition is of that composite type. For atopic dermatitis, no equivalent dedicated validation of UK Biobank phenotyping is known to us. The available support is indirect and comes from genome-wide association work, in which meta-analyses incorporating UK Biobank participants recover the canonical atopic dermatitis loci with effect directions consistent with clinically ascertained cohorts [26,27]. This is evidence of construct validity, not a quantification of case definition accuracy, and sensitivity and positive predictive value for L20 in this cohort remain unknown. Because atopic dermatitis is frequently managed entirely in primary care or through self-directed treatment, under-ascertainment is the more likely direction of error, which would attenuate rather than manufacture the contrasts reported here.

The coexistence group inherits the uncertainty of both definitions and adds one of its own: administrative data cannot distinguish true concurrence from sequential diagnostic revision, in which a patient receives one label and later the other as the clinical picture is reinterpreted. No date of first report field is available for L40 or L20 in this extraction, and the source-of-report fields record presence without retaining which source contributed it, so neither a temporal nor a multiple-source criterion could be constructed. This limitation is not resolvable within the present design.

The two analyses reported in Section 3.9 nonetheless bear on the plausibility of the group, and they converge. The deficit of coexistence relative to independence is incompatible with indiscriminate double coding and is consistent either with genuine partial mutual exclusivity, as the antagonism between the type 17 and type 2 axes would predict [10], or with diagnostic substitution. The polygenic evidence shows that the group carries authentic psoriasis genetic loading, attenuated by approximately half, while its atopic loading, as indexed by asthma and allergic rhinitis, is indistinguishable from that of isolated atopic dermatitis. The group is therefore atopically authentic and psoriatically diluted, which gives the misclassification an identifiable direction rather than leaving it undirected. The case for examining this population does not rest on certainty about its purity; it rests on the observation that patients carrying both diagnoses exist, present a genuine therapeutic dilemma because agents controlling one axis may aggravate the other [9], and have rarely been characterised systemically at scale.

### 4.2. Construction of the Comparator

The contrast between comparators is the methodological lesson of this study. Under the original design, the restricted control alone was free of obesity, myocardial infarction, atrial fibrillation and stroke, while those conditions remained present in the disease groups, and several of the biomarkers defining the apparent gradient are closely related to cardiometabolic health. Applying the exclusions symmetrically attenuated the metabolic contrasts by roughly half and abolished the monotonic ordering. What was reported as a severity gradient was substantially a property of the comparator.

This illustrates an asymmetry with general application. Unmeasured confounding, comparator construction and outcome misclassification tend to manufacture differences between groups; none of them can manufacture equivalence. Findings of equivalence and non-discrimination are therefore conservative with respect to precisely the biases that most threaten observational biomarker comparisons, whereas findings of ordination are not. In this study the two classes of finding pointed in opposite directions under scrutiny, and we have accordingly assigned them different weights.

### 4.3. The Circulating Compartment

Within the proteomic subset, the largest deviations in psoriasis were IL-6 and IL-17A, with type 2 markers close to zero in peripheral blood. It would be an error to read this as evidence against a type 2 signature in atopic dermatitis. IL-4, IL-13 and IL-31 circulate at or near the assay limit of quantification in unselected populations, and the compartment in which type 2 immunopathology is expressed is lesional skin. The proteomic subset here is also differentially ascertained, being three times more frequent in controls than in psoriasis, so selection of unknown direction cannot be excluded. These observations require replication in a proteomic sample without differential ascertainment and support no immunological conclusion on their own.

### 4.4. Limitations

The analysis is exploratory and cross-sectional, permitting neither causal nor temporal inference, and was performed with access to the outcomes, distinguishing it from the pre-registered confirmatory test it is intended to inform. The sample is a subselection of UK Biobank enriched for cardiometabolic disease, so the control, even when restricted, does not represent the general population.

Sex was included as a covariate in all adjusted models, but the analysis was not disaggregated by sex. This decision was taken because the study question concerns discrimination among diagnostic groups rather than modification of biomarker profiles by sex and because stratification would have reduced the coexistence group below the size at which the interaction tests retain useful power. Sex differences in the systemic expression of psoriasis and atopic dermatitis are plausible and remain an appropriate subject for dedicated analysis in a larger sample.

The cohort is elderly, with the median age between 73 and 75 years. It therefore captures prevalent, long-standing and frequently treated disease, and generalisation to earlier-onset populations, in whom disease activity and cardiometabolic accumulation differ, is not warranted.

Case definition relied on composite first-occurrence records without adjudication, with the specific consequences set out in Section 4.1. Smoking is captured by a binary indicator consistent with current rather than ever-smoking, and dietary pattern, alcohol intake and the Townsend deprivation index were not used, although income, physical activity and television viewing time were examined and did not alter the estimates. Glycoprotein acetyls were measured in 55.6% of the analytic sample and the Olink panel in approximately 29%, with differential coverage across groups. Finally, the size of the coexistence group limits the ability to detect subtle synergy, and the leucocyte indices anchoring the dominant factor are arithmetically redundant.

## 5. Conclusions

Within an elderly, cardiometabolically enriched UK Biobank subsample, the systemic haematological, biochemical and metabolomic biomarkers evaluated did not discriminate psoriasis, atopic dermatitis and their coexistence in this exploratory analysis. Effect magnitudes were negligible for the large majority of contrasts, formal equivalence was met for most and, under a symmetric comparator, for all contrasts examined, and coexistence showed no evidence of biomarker synergy. A severity gradient apparent under an asymmetric comparator did not survive symmetric exclusion and is therefore reported as a secondary and hypothesis-generating observation rather than as a finding. Polygenic evidence indicates that the coded groups carry the expected genetic architecture and that the coexistence group, while genetically genuine, is diluted with respect to psoriasis by approximately half.

These findings do not evaluate treatment response and support no therapeutic recommendation. What they support is the narrower observation that patients in all three dermatological groups carried a cardiometabolic burden exceeding that of a comorbidity-free comparator, consistent with existing guidance on cardiovascular risk assessment in chronic inflammatory skin disease [11,12]. Whether the absence of discrimination reflects shared systemic biology or the insensitivity of circulating markers to compartmentalised cutaneous inflammation remains open, and prospective, clinically adjudicated characterisation of patients with concurrent disease is the necessary next step.

## Figures and Tables

**Table 1:** Analytical architecture: mapping of method to target, estimand and decision criterion.

Target	Method and Estimand	Decision Criterion	Discriminates
Magnitude of difference	Cliff’s delta with bootstrap confidence intervals against the comparator	Negligible when |δ| < 0.147, equivalent to an area under the curve below 0.574	H0, H1 vs. H2
Affirmative overlap	Two one-sided tests against the margin |δ| = 0.147	Equivalence declared only when the entire confidence interval lies within the margin	H0 vs. H1, H2
Distributional location	Median quantile regression, repeated at the 0.75 and 0.90 quantiles	Effects confined to upper quantiles would indicate a subgroup phenomenon invisible at the median	Subgroup effects
Additivity vs. synergy	Interaction terms in quantile regression; MANOVA of the interaction	Synergy declared only if interaction terms survive false discovery rate control	H2 vs. H0, H1
Joint separation	PERMANOVA with Euclidean distance, with permutational test of dispersion	Interpreted by variance explained, not by the *p* value alone	H2 vs. H0, H1
Individual discrimination	Penalised logistic regression and random forest with cross-validation and permutation null	Meaningful discrimination requires performance materially above the permutation null	H2 vs. H0, H1
Discrete vs. continuous	Principal component analysis and Gaussian mixture modelling; adjusted Rand index	Correspondence between latent classes and diagnoses supports discrete phenotypes	H2 vs. H1, H0
Monotonic ordination	Dominant factor, then Jonckheere–Terpstra trend test across four ordered groups	A spectrum requires ordering that is monotonic and not attributable to the comparator	H1 vs. H0

H0, no discriminating systemic signature; H1, shared continuum with additive coexistence; H2, distinct phenotypes with synergy.

**Table 2:** Baseline characterisation by group, with the restricted control as reference.

Characteristic	Restricted Control	Isolated AD	Isolated Psoriasis	Coexistence
*n*	31,257	12,859	15,146	1068
Age, median (IQR), years	73 (66–79)	74 (66–79)	74 (67–79)	75 (68–80)
Female sex	56.9%	58.1%	49.8%	54.1%
BMI, median, kg/m^2^	26.2	26.7	27.5	27.9
Current smoking	10.2%	10.1%	14.9%	14.7%
Statin use	11.6%	15.4%	18.7%	20.4%
Obesity (E66)	0% *	13.7%	16.0%	19.2%
Myocardial infarction (I21)	0% *	5.1%	7.1%	6.7%
Atrial fibrillation (I48)	0% *	8.8%	11.3%	9.7%
GlycA, median	0.80	0.81	0.82	0.83
HDL, median, mmol/L	1.43	1.41	1.35	1.34
NLR, median	2.12	2.18	2.27	2.29
SIRI, median	0.92	0.98	1.05	1.06
Psoriasis PRS, mean, SD units	−0.195	−0.193	+0.457	+0.129

* Zero by definition of the restricted control. AD, atopic dermatitis; BMI, body mass index; GlycA, glycoprotein acetyls; HDL, high-density lipoprotein; IQR, interquartile range; NLR, neutrophil-to-lymphocyte ratio; PRS, polygenic risk score; SD, standard deviation; SIRI, systemic inflammation response index.

**Table 3:** Cliff’s delta (95% confidence interval) for selected markers, each clinical group versus the restricted control. Positive values indicate elevation relative to control.

Biomarker	AD vs. Control	Psoriasis vs. Control	Coexistence vs. Control
GlycA	+0.042 (+0.017, +0.069)	+0.124 (+0.099, +0.149)	+0.122 (+0.079, +0.174)
HDL	−0.027 (−0.054, −0.001)	−0.123 (−0.150, −0.098)	−0.118 (−0.167, −0.078)
LDL	−0.042 (−0.069, −0.014)	−0.059 (−0.086, −0.033)	−0.080 (−0.133, −0.030)
HbA1c	+0.029 (−0.001, +0.057)	+0.059 (+0.034, +0.087)	+0.127 (+0.081, +0.167)
ALT	+0.039 (+0.013, +0.063)	+0.105 (+0.080, +0.130)	+0.126 (+0.084, +0.165)
NLR	+0.049 (+0.021, +0.074)	+0.106 (+0.078, +0.132)	+0.111 (+0.071, +0.151)
MHR	+0.051 (+0.024, +0.078)	+0.141 (+0.116, +0.170)	+0.153 (+0.114, +0.194)
SIRI	+0.064 (+0.038, +0.088)	+0.139 (+0.114, +0.164)	+0.158 (+0.118, +0.194)
IL-6 (Olink) ^†^	—	+0.20 (+0.17, +0.23)	— (low *n*)
IL-17A (Olink) ^†^	—	+0.16 (+0.13, +0.19)	— (low *n*)

^†^ Olink proteins were available in a differentially ascertained subset (Section 2.3) and are reported as exploratory. AD, atopic dermatitis; ALT, alanine aminotransferase; GlycA, glycoprotein acetyls; HbA1c, glycated haemoglobin; HDL, high-density lipoprotein; LDL, low-density lipoprotein; MHR, monocyte-to-HDL ratio; NLR, neutrophil-to-lymphocyte ratio; SIRI, systemic inflammation response index.

**Table 4:** Decision matrix populated with the exploratory results.

Target	Observed Result	Interpretation	Favours
Between-group separation	PERMANOVA R^2^ = 0.6% (*p* = 0.005); multiclass AUC 0.57 against a permutation null of 0.49	Detectable separation of negligible magnitude; no individual-level discrimination	H0 and H1
Additivity vs. synergy	None of 16 interactions with q < 0.05; MANOVA Pillai trace 0.0005 (*p* = 0.003)	No relevant synergy; coexistence approximates the sum of its parts	H1, additive
Discrete vs. continuous	Gaussian mixture model with four classes by Bayesian information criterion; ARI 0.011; NMI 0.005	Latent classes do not correspond to clinical diagnoses	H0 and H1
Overlap by equivalence	38 of 48 equivalent against the restricted control and 47 of 48 against the broad control; under symmetric exclusion all contrasts examined met equivalence	Distributions indistinguishable for the large majority of markers, and for all markers once the comparator is symmetric	H0
Monotonic ordination	Medians ordered under the original comparator; ordering does not survive symmetric exclusion, coexistence falling below isolated psoriasis	The apparent gradient is substantially attributable to comparator construction	Inconclusive
Validity of definitions (post hoc)	PRS by group: control −0.195, AD −0.193, psoriasis +0.457, coexistence +0.129; arthropathic psoriasis 9.9% vs. 5.6%	Coded groups carry the expected polygenic architecture; coexistence genetically genuine but about half diluted	Supports classification
Frequency of coexistence (post hoc)	1068 observed against 1404 expected; odds ratio 0.72 (95% CI 0.68 to 0.77)	Recorded less often than chance predicts, incompatible with indiscriminate double coding	Supports classification

ARI, adjusted Rand index; AUC, area under the curve; NMI, normalised mutual information; PERMANOVA, permutational multivariate analysis of variance; PRS, polygenic risk score. H0, no discriminating systemic signature; H1, shared continuum with additive coexistence; H2, distinct phenotypes with synergy. H2 received no support from any criterion.

## Data Availability

Restrictions apply to the availability of these data. Data were obtained from UK Biobank under application 358626 and are available to bona fide researchers upon application to UK Biobank (https://www.ukbiobank.ac.uk) with the permission of UK Biobank. The authors are not permitted to redistribute individual-level data, and approved projects are conducted within the UK Biobank Research Analysis Platform. Every variable used in the analysis, with its source in the analytic dataset and its coverage, is listed in Table S1.

## Supplementary Materials

The following supporting information can be downloaded at: https://www.mdpi.com/article/10.3390/metabo16090632/s1. Figure S1: Participant flow diagram; Table S1: Inventory of the variables used in the analysis, with their source in the analytic dataset and their coverage; File S1: Completed STROBE checklist for cross-sectional studies.

## Abbreviations

The following abbreviations are used in this manuscript:

**Abbreviation**	**Definition**
AD	Atopic dermatitis
ALT	Alanine aminotransferase
ARI	Adjusted Rand index
AST	Aspartate aminotransferase
AUC	Area under the receiver operating characteristic curve
BIC	Bayesian information criterion
BMI	Body mass index
CI	Confidence interval
FDR	False discovery rate
GlycA	Glycoprotein acetyls
GMM	Gaussian mixture model
HbA1c	Glycated haemoglobin
HDL	High-density lipoprotein
ICD-10	International Classification of Diseases, 10th revision
IQR	Interquartile range
LDL	Low-density lipoprotein
LLR	Leucocyte-to-lymphocyte ratio
MANOVA	Multivariate analysis of variance
MHR	Monocyte-to-HDL ratio
MLR	Monocyte-to-lymphocyte ratio
NLR	Neutrophil-to-lymphocyte ratio
NMI	Normalised mutual information
NPX	Normalised protein expression
PC	Principal component
PERMANOVA	Permutational multivariate analysis of variance
PERMDISP	Permutational analysis of multivariate dispersions
PLR	Platelet-to-lymphocyte ratio
PRS	Polygenic risk score
SD	Standard deviation
SII	Systemic immune–inflammation index
SIRI	Systemic inflammation response index
STROBE	Strengthening the Reporting of Observational Studies in Epidemiology
TOST	Two one-sided tests
VLDL	Very-low-density lipoprotein
