# Supplementary material for: Systemic Biomarkers Do Not Discriminate Psoriasis, Atopic Dermatitis and Their Coexistence: An Equivalence-Based Cross-Sectional Analysis of 160,782 UK Biobank Participants
Source: Metabolites. 2026 Aug 30;16(9):632. doi: 10.3390/metabo16090632 (PMC13609600; doi:10.3390/metabo16090632)
Supplement: Supplementary file 1 [file metabolites-16-00632-s001.zip › File_S1_STROBE_checklist.pdf]

File S1. STROBE Statement

Manuscript metabolites-4478726 · Systemic Biomarkers Do Not Discriminate Psoriasis, Atopic Dermatitis and Their Coexistence: An Equivalence-Based Cross-Sectional Analysis of 160,782 UK Biobank Participants · Metabolites (MDPI)

Item	Topic	Recommendation	Location in the manuscript
**Title and abstract**			
1	Title and abstract	(a) Indicate the study design with a commonly used term in the title or the abstract.	Title, which states an equivalence-based cross-sectional analysis; Abstract, Methods sentence.
		(b) Provide in the abstract an informative and balanced summary of what was done and what was found.	Abstract, structured under Background/Objectives, Methods, Results and Conclusions; Highlights.
**Introduction**			
2	Background/rationale	Explain the scientific background and rationale for the investigation being reported.	Section 1, paragraphs 1 to 3.
3	Objectives	State specific objectives, including any prespecified hypotheses.	Section 1, final paragraph, where the three competing architectures H0, H1 and H2 are stated; Section 2.7, where each is bound to a decision criterion.
**Methods**			
4	Study design	Present key elements of study design early in the paper.	Section 2.1.
5	Setting	Describe the setting, locations, and relevant dates, including periods of recruitment, exposure, follow-up, and data collection.	Sections 2.1 and 2.2 (UK Biobank, application 358626, linkage to national health datasets); Section 4.4 for the age structure of the cohort.
6	Participants	(a) Give the eligibility criteria, and the sources and methods of selection of participants.	Section 2.2, third and fourth paragraphs: derivation of 160,782 participants from 501,979, the four mutually exclusive groups, and the restricted control (n = 31,257). Figure S1 gives the flow diagram.
7	Variables	Clearly define all outcomes, exposures, predictors, potential confounders, and effect modifiers. Give diagnostic criteria, if applicable.	Section 2.2 for the ICD-10 case definitions (L40, L20); Section 2.3 for the three biomarker layers; Section 2.4 for the adjustment set. Table S1 inventories every variable with its source and coverage.
8*	Data sources/ measurement	For each variable of interest, give sources of data and details of methods of assessment (measurement). Describe comparability of assessment methods if there is more than one group.	Section 2.2 for outcome ascertainment and the four contributing sources; Section 2.3 for the haemato-inflammatory, biochemical-metabolomic and proteomic panels. Assessment is identical across groups, since all measurements derive from the same centralised UK Biobank protocols.
9	Bias	Describe any efforts to address potential sources of bias.	Section 2.2, fourth paragraph, for the restricted control; Section 2.4 for statin use as a masking factor; Section 2.7, fourth paragraph, for the

Item	Topic	Recommendation	Location in the manuscript
			asymmetry of the criteria with respect to bias; Section 2.9 for the sensitivity analyses, including symmetric cardiometabolic exclusion.
10	Study size	Explain how the study size was arrived at.	Section 2.2, third paragraph. No a priori sample size calculation was performed; the analytic sample was determined by the availability of the index conditions and of biomarker measurements. The smallest interaction effect detectable at 80% power is reported in Section 3.11, which bounds any undetected synergy.
11	Quantitative variables	Explain how quantitative variables were handled in the analyses. If applicable, describe which groupings were chosen and why.	Section 2.5: standardisation to z-scores on the median and interquartile range of the restricted control, winsorisation at plus or minus five standard deviations, and treatment of missing data.
12	Statistical methods	(a) Describe all statistical methods, including those used to control for confounding.	Sections 2.7 and 2.8: Cliff's delta with the negligibility threshold $\|\delta\| < 0.147$, two one-sided tests for equivalence, median quantile regression with interaction terms, MANOVA, PERMANOVA, penalised logistic regression and random forest, principal component analysis and Gaussian mixture modelling, and the Jonckheere–Terpstra trend test. Table 1 maps each method to its target and decision criterion.
		(b) Describe any methods used to examine subgroups and interactions.	Section 2.8 for the psoriasis by atopic dermatitis interaction; Section 2.9 for its re-examination at the 0.75 and 0.90 quantiles.
		(c) Explain how missing data were addressed.	Section 2.5 for complete-case analysis with the effective number reported; Section 2.9 and Section 3.11 for multiple imputation by chained equations as a confirmatory sensitivity analysis.
		(d) If applicable, describe analytical methods taking account of sampling strategy.	Not applicable. No complex sampling design was used; the analytic sample is a subselection of an existing cohort, with the consequences stated in Section 4.4.
		(e) Describe any sensitivity analyses.	Section 2.9, and the executed results in Section 3.11: equivalence margin, PERMDISP, distributional location, treatment exclusion, missing data, collinearity, symmetric cardiometabolic exclusion and extended covariate adjustment.
**Results**			

Item	Topic	Recommendation	Location in the manuscript
13*	Participants	(a) Report numbers of individuals at each stage of the study.	Section 2.2, third and fourth paragraphs; Table 2, first row; Figure S1.
		(b) Give reasons for non-participation at each stage.	Not applicable in the conventional sense, since no new recruitment was undertaken. Reductions in the analysable number arise from biomarker coverage, reported per panel in Section 2.3 and per variable in Table S1.
		(c) Consider use of a flow diagram.	Figure S1.
14*	Descriptive data	(a) Give characteristics of study participants and information on exposures and potential confounders.	Section 3.1 and Table 2.
		(b) Indicate number of participants with missing data for each variable of interest.	Section 2.3 for panel coverage, including the approximately 29% coverage of the proteomic layer; Table S1 for coverage of every variable.
		(c) Summarise follow-up time.	Not applicable. The design is cross-sectional and no follow-up was undertaken (Section 2.1).
15*	Outcome data	Report numbers of outcome events or summary measures.	Section 3.1 and Table 2 for the group medians and prevalences; Section 3.2 and Table 3 for the biomarker effect sizes; Figure 1.
16	Main results	(a) Give unadjusted estimates and, if applicable, confounder-adjusted estimates and their precision. Make clear which confounders were adjusted for and why they were included.	Table 3 for unadjusted Cliff's deltas with 95% confidence intervals against the restricted control; Sections 3.4 and 3.11 for the covariate-adjusted quantile regression estimates; Section 2.4 states the adjustment set and the reason for each term.
		(b) Report category boundaries when continuous variables were categorised.	Section 2.5. Biomarkers were analysed on a continuous scale and were not categorised. The negligibility threshold $\|\delta\| < 0.147$ and the equivalence margins of 0.11, 0.147 and 0.20 are decision thresholds on the effect-size scale, defined in Sections 2.8 and 2.9.
		(c) If relevant, consider translating estimates of relative risk into absolute risk for a meaningful time period.	Not applicable. The estimands are ordinal effect sizes and distributional contrasts, not risk measures. The algebraic relation of Cliff's delta to the area under the receiver operating characteristic curve is given in Section 2.8 to aid interpretation.
17	Other analyses	Report other analyses done, e.g. analyses of subgroups and interactions, and sensitivity analyses.	Sections 3.4 to 3.9 for the pre-specified analyses; Section 3.11 for the sensitivity analyses. The three post hoc analyses are identified as such in Section 2.7, final paragraph, and reported in Sections 3.9 and 3.11.
**Discussion**			

Item	Topic	Recommendation	Location in the manuscript
18	Key results	Summarise key results with reference to study objectives.	Section 3.10 for the decision synthesis against the three architectures, with Table 4; Section 4, first and third paragraphs.
19	Limitations	Discuss limitations of the study, taking into account sources of potential bias or imprecision. Discuss both direction and magnitude of any potential bias.	Section 4.4, with the direction of bias stated explicitly in Section 2.7, fourth paragraph, and in Section 4.2; Section 4.1 for the case definitions and Section 4.3 for the proteomic layer.
20	Interpretation	Give a cautious overall interpretation of results considering objectives, limitations, multiplicity of analyses, results from similar studies, and other relevant evidence.	Section 4, second paragraph, which states what the findings do not establish; Sections 4.1 to 4.3; Section 5.
21	Generalisability	Discuss the generalisability (external validity) of the study results.	Section 4.4, third paragraph, on the elderly and cardiometabolically enriched nature of the subsample.
**Other information**			
22	Funding	Give the source of funding and the role of the funders for the present study and, if applicable, for the original study on which the present article is based.	Funding statement in the back matter; UK Biobank application number 358626 stated in Section 2.1 and in the Acknowledgments.

Notes

1. An explanation and elaboration article discusses each checklist item and gives methodological background and published examples of transparent reporting. The STROBE checklist is best used in conjunction with that article, which is freely available at https://www.strobe-statement.org.

2. Items marked with an asterisk should, in cohort and case-control studies, be given separately for exposed and unexposed groups or for cases and controls. In this cross-sectional study the corresponding information is given separately for each of the four diagnostic groups.

3. Locations refer to the numbered sections of the manuscript rather than to page numbers, since pagination may change during production. Section numbering is stable across the accepted text.
