# Supplementary material for: Systemic Biomarkers Do Not Discriminate Psoriasis, Atopic Dermatitis and Their Coexistence: An Equivalence-Based Cross-Sectional Analysis of 160,782 UK Biobank Participants
Source: Metabolites. 2026 Aug 30;16(9):632. doi: 10.3390/metabo16090632 (PMC13609600; doi:10.3390/metabo16090632)
Supplement: Supplementary file 1 [file metabolites-16-00632-s001.zip › Legenda Figure S1.pdf]

Figure S1. Derivation of the analytic subsample and constitution of the study groups. Of the 501,979 participants in the UK Biobank cohort, 341,197 were excluded for having neither GLP-1 receptor assay data nor a record of any of the six index conditions, leaving an analytic subsample of 160,782. This subsample was partitioned into four mutually exclusive groups by the presence or absence of first-occurrence records for psoriasis (L40) and atopic dermatitis (L20): control, defined by the absence of both codes; isolated atopic dermatitis; isolated psoriasis; and coexistence, defined by the presence of both codes in the same individual. Because the subsample is enriched for cardiometabolic disease, a restricted control additionally free of obesity (E66), myocardial infarction (I21), atrial fibrillation (I48) and stroke (I64) was adopted as the primary comparator, and the broad control of all 131,709 participants without L40 or L20 was retained as a sensitivity analysis. The lower row shows the sensitivity analysis in which the same four cardiometabolic exclusions are applied symmetrically to all groups rather than to the control alone, reported in Section 3.11. Codes are three-character categories of the International Classification of Diseases, tenth revision.
