# Supplementary material for: Systemic Biomarkers Do Not Discriminate Psoriasis, Atopic Dermatitis and Their Coexistence: An Equivalence-Based Cross-Sectional Analysis of 160,782 UK Biobank Participants
Source: Metabolites. 2026 Aug 30;16(9):632. doi: 10.3390/metabo16090632 (PMC13609600; doi:10.3390/metabo16090632)
Supplement: Supplementary file 1 [file metabolites-16-00632-s001.zip › Table_S1_Variable_Inventory.pdf]

Supplementary Table S1

metabolites-4478726

Table S1. Inventory of the variables used in the analysis, with their source in the analytic dataset and their coverage.

Every variable entering any analysis reported in the main text is listed, grouped by domain. The first column gives the name used in the manuscript and the second the corresponding variable in the analytic dataset, which is either a field distributed by UK Biobank or, where indicated as derived, a quantity computed by the authors from the listed components. Coverage is the proportion of the 160,782 participants of the analytic subsample with a non-missing value for that variable, and is reported because it varies substantially across domains: it approaches completeness for demography and the haematological panel, falls to approximately 55 per cent for the nuclear magnetic resonance metabolomics subset that includes glycoprotein acetyls, and falls further to approximately 29 per cent for the Olink inflammatory proteins. Coverage of the Olink panel is additionally uneven across the four clinical groups, ranging from 10.8 per cent in psoriasis to 32.3 per cent in the control group, which is why that arm is treated as exploratory throughout and excluded from the decision synthesis; the group-specific figures are given in Section 2.3 of the main text. Variables listed with coverage below the threshold adopted for the primary models were either restricted to sensitivity analyses or not used, as stated in the Notes column.

Variable as used in the manuscript	Source variable in the analytic dataset	Coverage	Notes
**Identification and demography**			
Age	Age	100%	Attained age, not age at recruitment
Sex	Sex	100%	Genetic sex not used
Ethnicity	Ethnic background | Instance 0	99.8%	Dichotomised as white versus other
Body mass index	Body mass index (BMI) | Instance 0	97.2%	Instance 0
Year of birth	Year of birth	100%	Not used in models
**Group-defining and index conditions (first-occurrence fields, Category 1712)**			
Psoriasis (L40)	Source of report of L40 (psoriasis)	10.1%	Three-character aggregation, subsumes L40.0 to L40.9
Atopic dermatitis (L20)	Source of report of L20 (atopic dermatitis)	8.7%	Subsumes L20.0, L20.8, L20.9
Obesity (E66)	Source of report of E66 (obesity)	33.0%	Restricted-control exclusion
Myocardial infarction (I21)	Source of report of I21	15.9%	Restricted-control exclusion
Atrial fibrillation (I48)	Source of report of I48	26.9%	Restricted-control exclusion
Stroke (I64)	Source of report of I64	6.7%	Restricted-control exclusion
Arthropathic psoriasis (L40.5)	L40.5 indicator	1.0%	Used only in the validity analysis, Section 3.9
Psoriatic arthropathy (M07)	Source of report of M07	1.0%	Used only in the validity analysis
Asthma (J45)	Source of report of J45	18.0%	Atopy proxy, validity analysis
Allergic rhinitis (J30)	Source of report of J30	11.1%	Atopy proxy, validity analysis
**Haematology and derived inflammatory indices (Category 100081)**			
White blood cell count	White blood cell (leukocyte) count	95.3%	Component of LLR and SII
Lymphocyte count	Lymphocyte count	95.2%	Common denominator of all ratios

Variable as used in the manuscript	Source variable in the analytic dataset	Coverage	Notes
Monocyte count	Monocyte count	95.1%	Component of MLR, MHR, SIRI
Neutrophil count	Neutrophil count	95.2%	Component of NLR, SII, SIRI
Platelet count	Platelet count	95.3%	Component of PLR and SII
NLR	NLR	95.2%	Neutrophils / lymphocytes
PLR	PLR	95.2%	Platelets / lymphocytes
MLR	MLR	95.1%	Monocytes / lymphocytes
LLR	LLR	95.2%	Leucocytes / lymphocytes
MHR	MHR	83.4%	Monocytes / HDL; coverage limited by HDL
SII	SII	95.2%	Platelets x neutrophils / lymphocytes
SIRI	SIRI	95.1%	Neutrophils x monocytes / lymphocytes
**Clinical biochemistry (Category 17518) and NMR metabolomics (Category 220)**			
Total cholesterol	Cholesterol	55.4%	
LDL cholesterol	LDL direct	55.4%	
VLDL cholesterol	VLDL cholesterol	55.4%	NMR panel
HDL cholesterol	HDL cholesterol	85.8%	
Triglycerides	Triglycerides	93.6%	
Alanine aminotransferase	Alanine aminotransferase	93.6%	
Aspartate aminotransferase	Aspartate aminotransferase	93.3%	
Glycated haemoglobin	Glycated haemoglobin (HbA1c)	93.2%	
Glycoprotein acetyls	Glycoprotein Acetyls	55.5%	NMR metabolomics subset
**Olink inflammatory proteins (Category 1839)**			
IL-6, IL-12, IL-18	IL6; IL12; IL18	28.7%	Differential coverage by group, see Section 2.3
IL-17A, IL-17C, IL-17F	IL17A; IL17C; IL17F	28.7%	Exploratory arm
IL-4, IL-13, IL-31	IL4; IL13; IL31	28.7%	Type 2 markers, near limit of quantification
FCER1A, FCER2	FCER1A; FCER2	26.9 to 27.9%	Exploratory arm
**Genetics**			
Psoriasis polygenic risk score	Standard PRS for psoriasis (PSO)	97.1%	Standard PRS release; used only in the post hoc validity analysis
**Covariates and lifestyle**			
Smoking indicator	Smoking status | Instance 0	11.7%	Derived binary; prevalence consistent with current smoking

Variable as used in the manuscript	Source variable in the analytic dataset	Coverage	Notes
Statin use	Treatment/medication code | Instance 0	80.7%	Identified by text analysis of medication codes
Immunomodulatory or biologic therapy	Treatment/medication code | Instance 0	80.7%	Identified by text analysis; sensitivity analysis
Household income	Average total household income before tax	84.9%	Extended adjustment, Section 3.11
Physical activity	Number of days/week of moderate physical activity	93.4%	Extended adjustment
Television viewing time	Time spent watching television (TV)	96.5%	Extended adjustment
Alcohol intake frequency	Alcohol intake frequency	28.3%	Not used; coverage insufficient

Derived indicates a variable computed by the authors from the listed components rather than distributed directly by UK Biobank. Group-defining and index conditions were ascertained from the UK Biobank first-occurrence fields, which harmonise hospital inpatient, primary care, death register and self-reported records at the three-character ICD-10 level. HDL, high-density lipoprotein; ICD-10, International Classification of Diseases, 10th revision; LDL, low-density lipoprotein; LLR, leucocyte-to-lymphocyte ratio; MHR, monocyte-to-HDL ratio; MLR, monocyte-to-lymphocyte ratio; NLR, neutrophil-to-lymphocyte ratio; NMR, nuclear magnetic resonance; PLR, platelet-to-lymphocyte ratio; PRS, polygenic risk score; SII, systemic immune-inflammation index; SIRI, systemic inflammation response index; VLDL, very-low-density lipoprotein.
